# Can the salivary urea and stimulated saliva concentration be a marker of periodontal diseases in opioid users? A case-control study

**DOI:** 10.1016/j.heliyon.2023.e17093

**Published:** 2023-06-28

**Authors:** Parvin parvaei, Marzie eydzadeh, Freshteh Osmani

**Affiliations:** a Dentistry Clinical Research Development Center, Birjand University of Medical Sciences, Birjand, Iran; b Student Research Committee, Birjand University of Medical Sciences, Birjand, Iran; c Infectious Diseases Research Center, Birjand University of Medical Sciences, Birjand, Iran

**Keywords:** Periodontitis, Salivary urea, Opioids, Stimulated saliva

## Abstract

**Introduction:**

& Aim: Microbial plaque is the primary cause of periodontal diseases, and smoking and opioid addiction can accelerate microbial plaque formation and disease progression. Adequate saliva flow and salivary urea concentration are important parameters for a healthy periodontium. In this study, the relationship between Periodontal Diseases and the History of opioid addiction was investigated by measuring the Salivary Urea and Stimulated Saliva Concentration.

**Materials & methods:**

This case-control study was conducted on 240 patients (120 cases and 120 controls) in 2021 referred to addiction treatment centers and the dental clinic in Iran, Birjand. The control and case groups were matched in terms of age. Demographic, base data, and clinical examination results were collected by a checklist. Data were analyzed using SPSS 19 and one-way ANOVA and chi-square tests. *P*-value <0.05 was considered as the significance level.

**Results:**

Periodontitis severity was significantly higher in the case group than in the control group (*P*-value = 0/000). Salivary urea concentration significantly increased in both case and control groups with an increase in periodontitis severity (*P*-value = 0/003 in the case group and *P*-value = 0/000 in the control group), but there was no significant relationship between the stimulated saliva flow rate and the severity of periodontitis in these two groups (*P*-value>0.05).

**Conclusion:**

Following the use of opioids, the flow of saliva decreases, and with the exacerbation of the periodontal disease, the concentration of urea in saliva increases. Therefore, it seems that the analysis of saliva parameters, including urea concentration, can be useful for the diagnosis of periodontal disease, and saliva urea concentration is not directly related to opioid use.

## 1 Introduction

In addition to social and economic problems, addiction endangers human health, including oral health. Opioids cause oral dryness, discoloration of oral tissues and periodontal diseases [1].

The primary cause of periodontal diseases is microbial plaque, and factors such as lack of oral hygiene, smoking and opioid addiction. In addition, oral dryness and tooth decay can accelerate microbial plaque formation and disease progression [2]. A higher prevalence of decay and periodontitis in drug addicts is closely related to decreased saliva secretion, a high carbohydrate diet, and poor oral health [3,4].

The saliva volume depends on various factors such as stimulation, circadian rhythm, diet, age, drugs and hydrogen ion concentration, and these factors can change due to pathological conditions such as periodontitis [[5], [6], [7]]. Clinical periodontal measurements such as pocket depth measurement, bleeding on probing (BOP) and attachment loss rate are indicative of the past disease rather than current disease activity [8]. Different salivary biomarkers are used for screening and predicting early changes in periodontal tissue because they allow the rapid screening. Considering the importance of saliva in the formation of oral biofilm and host immunity, secreted saliva can play an important role in the development and progression of periodontal diseases [[9], [10], [11], [12], [13]].

A sufficient salivary flow rate is a prerequisite for oral health, and the salivary urea concentration is an important parameter for tooth and gingiva health [14,15]. The salivary pH decreases and the risk of dental plaque formation increases due to opioid-induced oral dryness [16]. The urease enzyme of microbial plaque bacteria hydrolyzes salivary urea into ammonia and carbon dioxide (CO_2_), which are very destructive to periodontal tissues; therefore, the analysis of salivary urea concentration reflects the disease activity and severity [17].

Although several epidemiological studies have shown the relationship between periodontitis and opioid addiction, there is little information about the mechanisms of occurrence and severity of periodontitis in opioid addicts. Therefore, the salivary urea concentration as one of the salivary biomarkers affecting periodontium was investigated in the present study to evaluate its effect on the periodontal status of opioid addicts and the control group.

## 2 Materials and methods

### 2.1 Type of study and target population

In the present case-control study, 120 people were assigned to the case group and were selected from patients referred to addiction treatment centers and 120 people were also assigned to the control group and were selected from patients who visited the Periodontology Department of the Faculty of Dentistry and Samen Dental Clinic in Birjand in 2021.

### 2.2 Inclusion and exclusion criteria

Inclusion criteria included the mean age of 20–40 years and addiction to opioids in the case group for at least one year. Exclusion criteria also included patients with underlying diseases such as diabetes, immunodeficiency, leukemia, hepatitis, Human Immunodeficiency Virus (HIV), upper respiratory infection, necrotizing ulcerative gingivitis (NUG) mucosal lesions and invasive periodontitis or undergoing periodontitis treatment, a history of regular use of mouthwashes and taking antibacterial or anti-inflammatory drugs in the last three months and taking vitamin supplements, a history of chemotherapy and radiotherapy that caused xerostomia (17).

### 2.3 Data collection

Data was collected by a checklist which included demographic, baseline data and clinical exam results. Demographic information included age, gender, place of residence, occupation, level of education, and base data included opioid addiction, type of opioid (methadone, opium extract, and opium, methamphetamine), route of abuse, (smoking, non-smoking), and duration of opioid addiction (1–2 years, 2–4 years, more than 4 years).

### 2.4 Periodontal status assessment

Then periodontal tissue examination was carried out by measuring clinical attachment loss (CAL) at six sites and recorded on each tooth, except third molars using William's probe and the results were recorded in the information form and calibrated by a senior dental student (17). According to the degree of attachment loss, the samples were divided into four groups: no periodontitis, mild periodontitis (CAL = 1–2 mm), moderate periodontitis (CAL = 3–4 mm), and severe periodontitis (CAL = 5 mm and more) [18].

### 2.5 Saliva collection

To collect stimulated saliva, each participant was asked to chew a piece of unflavored rubber dam for 1 min to collect his/her saliva in a calibrated falcon tube between 9.00 a.m. and 12.00 p.m. Saliva samples were refrigerated at 4 °C for salivary urea (17). The stimulated salivary concentration was observed and recorded on the calibrated falcon. Salivary samples were analyzed to assess the salivary urea (Roche Diagnostics Pvt Ltd®, India).

### 2.6 Ethical considerations

This study was approved by the ethics committee of the Birjand University of Medical Sciences with the ethics code number IR.BUMS.REC.1399.300. Before participating in the study, patients completed an informed consent form. In addition, they were assured of the confidentiality of their information.

### 2.7 Statistical analysis

The results of the evaluations were recorded in the information form and entered into SPSS ver. 19. Data analysis was carried out using Chi-square and one way ANOVA tests and a *P*-value<0.05 was considered as the significance level.

## 3 Results

**Fig. 1:**
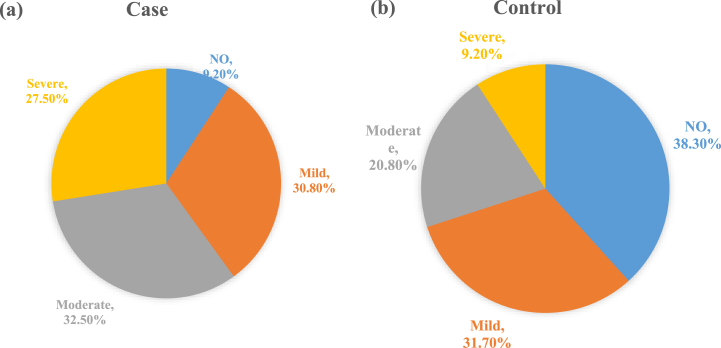
Distribution of degrees of periodontitis in the case group(a) and control group(b).

**Fig. 2:**
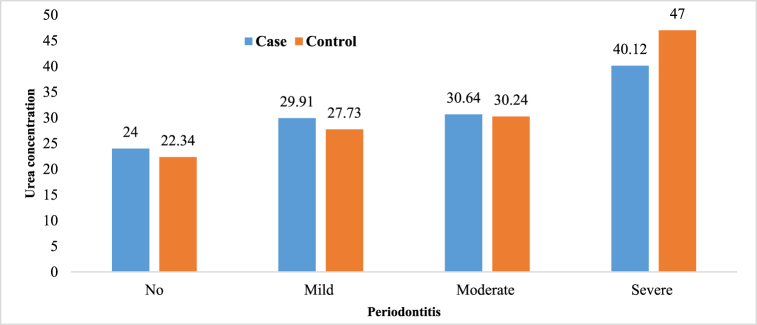
The mean salivary urea concentration in the study groups with different degrees of periodontitis.

**Fig. 3:**
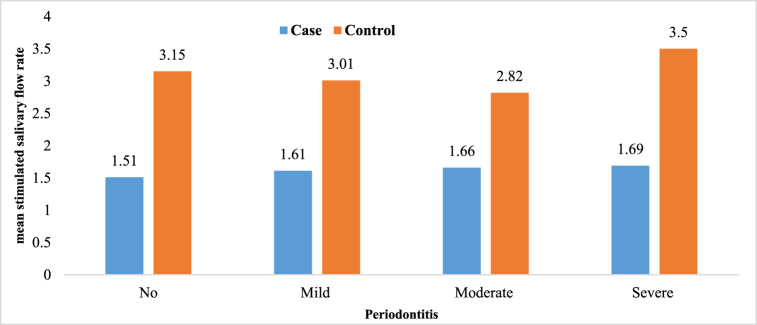
The mean stimulated saliva flow rate in the control group with different degrees of periodontitis.

Prevalence of higher degrees of periodontitis is higher in the case group; moderate periodontitis 32.5 vs. 20.8% and severe periodontitis 27.5 vs. 9.2% (Fig. 1). The results of the One way ANOVA test showed that salivary urea concentration increased significantly in both case and control groups with an increase in periodontitis severity (*P*-value<0.05), but there was no significant relationship between the stimulated saliva flow rate and the severity of periodontitis in these two groups (*P*-value>0.05). More details was presented in Table 1, Fig. 2, Fig. 3.

**Table 1:** Salivary urea concentration and the stimulated saliva flow rate in the study groups with different degrees of periodontitis.

Group			Urea (mg/ml)		Saliva (ml/min)	
		No (%)	Mean ± SD	P value	Mean ± SD	P value
	No periodontitis	11 (9.2%)	24.00 ± 9.4		1.51 ± 0.79	
**Case**	Mild periodontitis	37 (30.8%)	29.91 ± 14.7		1.61 ± 0.81	0.953
	Moderate periodontitis	39 (32.5%)	30.64 ± 11.1	0.003	1.66 ± 0.90	
	Severe periodontitis	33 (27.5%)	40.12 ± 17.7		1.69 ± 1.1	
	Total	120 (100%)	32.41 ± 14.9		1.64 ± 0.92	
**Control**	No periodontitis	46 (38.3%)	22.34 ± 9.70		3.15 ± 0.69	0.062
	Mild periodontitis	38 (31.7%)	27.73 ± 12.40		3.01 ± 0.74	
	Moderate periodontitis	25 (20.8%)	30.24 ± 10.00		2.82 ± 0.70	
	Severe periodontitis	11 (9.2%)	47.00 ± 28.99	0.000	3.50 ± 0.86	
	Total	120 (100%)	27.95 ± 14.90		3.07 ± 0.74

The results of the Chi-square test showed that the periodontitis severity in drug addicts is significantly higher than in the control group (*P*-value<0.05) but there was no significant relationship between periodontitis severity with the route of abuse (smoking-non-smoking), type of opioid (methadone, opium, methamphetamine) and the duration of addiction (*P*-value>0.05) (Table 2).

**Table 2:** The relationship between periodontitis and drug addiction.

Group		periodontitis				p-value
		No (No (%))	Mild (No (%))	Moderate (No (%))	Severe (No (%))	
Opioid addiction use	Yes (No:120)	11 (9.2%)	37 (30.8%)	39 (32.5%)	33 (27.5%)	0.000
	No (No:120)	46 (38.3%)	38 (31.7%)	25 (20.8%)	11 (9.2%)	
	Total (No:240)	57 (23.8%)	75 (31.2%)	64 (26.7%)	44 (18.3%)	
Duration of addiction	1–2 years (No:17)	3 (17.6%)	2 (11.8%)	5 (29.4%)	7 (41.2%)	
	2–4 years (No:20)	3 (15.0%)	7 (35.0%)	6 (30.0%)	4 (20.0%)	0.317
	Above 4 years (No:83)	5 (6.0%)	28 (33.7%)	28 (33.7%)	22 (26.5%)	
type of opioid	Methadone (No:84)	8 (9.5%)	25 (29.8%)	27 (32.1%)	24 (28.6%)	
	Opium (No:32)	3 (9.4%)	10 (31.3%)	11 (34.4%)	8 (25.0%)	0.981
	Methamphetamine (No:4)	0 (0.0%)	2 (50.0%)	1 (25.0%)	1 (25.0%)	
Route of abuse	Smoking (No:35)	3 (8.6%)	12 (34.3%)	12 (34.3%)	8 (22.9%)	
	Non-smoking (No:85)	8 (9.4%)	25 (29.4%)	27 (31.8%)	25 (29.4%)	0.984

## 4 Discussion

Different salivary biomarkers are used for screening and predicting early changes in periodontal tissues and for determining the effectiveness of treatment because they enable rapid screening as well as an accurate and valid assessment of periodontitis severity [[19], [20], [21]].

In the present study, there was no significant relationship between the stimulated saliva flow rate and the periodontitis severity in both case and control groups; however, the saliva flow rate increased in the case group with an increase in the periodontitis severity, but this relationship was not significant. Idrees et al. (2018) and Rajesh et al. (2015) found that unstimulated saliva secretion increased significantly with increasing severity of periodontitis [22,23]. Hirotomi et al. study (2006) found no significant relationship between the severity of periodontitis and unstimulated saliva flow [24]. The saliva flow rate was higher in the periodontitis group due to its inflammatory effect that can stimulate the salivary nerve. This protective salivary effect can itself stimulate the body's defense mechanism against the inflammatory process [23].

The present study revealed a significant increase in the saliva urea concentration of both groups with an increase in the periodontitis severity, which is consistent with the results of the study by Patil et al. (2020). Patil et al. reported a statistically significant increase in urine analysis (UA) levels in smokers with severe periodontitis compared to other groups (non-smokers, non-smokers with gingivitis, and smokers with moderate periodontitis) [17]. Bezerra et al. (2010) showed increased urea levels in patients with chronic periodontitis. Urea is hydrolyzed into ammonia and CO_2_ by bacterial urease enzymes, which is an important way to increase pH in the oral cavity. Ammonia, which is potentially cytotoxic, increases the epithelium permeability to other antigenic and toxic substances and plays a fundamental role in the initiation of periodontitis [25]. Luke et al. (2015) stated that the salivary blood urea nitrogen (BUN) level significantly increased in patients with chronic periodontitis compared to people with gingivitis and healthy people. Therefore, salivary urea concentration measurement can be useful for diagnosis, prognosis and evaluation of periodontitis treatment outcomes [26].

Gaál Kovalčíková (2019) showed that periodontitis increases the salivary urea concentration, but it is most likely not a result of blood contamination. In this study, blood was artificially added to the saliva of a group of patients, and tests showed that salivary urea increased only with very high levels of blood contamination (more than 2.5% salivary blood concentration), which was not normally present in the patients [27].

The results revealed that periodontitis was significantly more severe in drug addicts compared to the control group, which is consistent with the study by Saini et al. (2013). Saini reported a high prevalence of periodontitis with severe plaque build-up, which is characterized by attachment loss. Most addicts have a high rate of plaque accumulation, deposits due to the neglect of oral hygiene, dry mouth, and changes in the microbial profile. The use of drugs such as opioids leads to the suppression of pain responses and causes the patient to ignore the symptoms of tooth decay, periodontal diseases and limited access to dental care [2]. In the Shekarchizadeh et al. study (2019) of 217 opium (70%) and crystalline heroin (22%) users, 24.4% were edentulous. Older patients (p < 0.001) and patients with lower socioeconomic status (p = 0.01) had higher DMFT scores. None of the dentate patients had a healthy periodontium. Older participants (p = 0.02) and those who started drug abuse at a younger age (p = 0.01) were more likely to develop periodontal pockets [28].

The result of a meta-analysis study showed that drug type was associated with periodontal disease (OR 1.44; 95% CI 0.8–2.6) and pooled estimates showed that type of drug used increased the odds of the number of decayed, missed and filled teeth (DMFT) (OR 4.11; 95% CI 2.07–8.15) respectively [29]. Also, the result of Rotemberg et al. [30] showed that in addict patients, between the ages between 15 and 24, the DMF Index was 5.31, while in the ages between 25 and 35, it was 11.27. The periodontal survey showed that 65% of the participants suffered from gingivitis and 18% from periodontitis. Therefore, first-level health services should take special prevention and early detection measures when treating patients who are drug addicts.

There was no significant relationship between periodontitis and the type of opioid (smoking-non-smoking), routes of abuse (methadone, opium extract, opium, and methamphetamine) and the duration of addiction. Ye et al. (2014) stated that community periodontal index (CPI) scores were higher in people with four-year opioid abuse than those with shorter addiction durations [31]. In a longitudinal study, Kibayashi et al. (1999 until 2003) also stated that the number, duration and dose of smoking were related to the periodontitis progression [32], which is not consistent with the results of the present study. This discrepancy could be due to differences in the level of oral hygiene, type of drugs or criteria used to measure the periodontitis severity. On the other hand, more importantly, some of the reported deleterious effects of smoking on periodontal tissues have been reported to be reversible upon participation in smoking-cessation programs. Therefore, clinicians should strongly advise smokers to enrol in cessation strategies, even temporarily, to improve the overall outcome [33]. The Ravidà et al. study results showed that it took almost 15 years of smoking cessation for the risk of tooth loss due to periodontitis among former smokers to reach the level of never-smokers [34].

### 4.1 Limitation

During the collection of saliva, saliva could flow out of the falcon in 1 min, so the amount of stimulated saliva was measured approximately.

Also at first, our goal was to investigate salivary urea in opium, marijuana, hashish, heroin, amphetamine, methamphetamine, and cocaine users. Since marijuana users did not go to the addiction treatment center for withdrawal, and cocaine, heroin, and hashish use was rare in Birjand city, the study samples were selected from opium, methadone, and methamphetamine users.

## 5 Conclusion

Following the use of opioids, the flow of saliva decreases, and with the exacerbation of the periodontal disease, the concentration of urea in saliva increases; therefore, it seems that the analysis of saliva parameters, including urea concentration, can be useful for the diagnosis of periodontal disease, and saliva urea concentration is not directly related to opioids use.

## Author contribution statement

Marzie Eydzadeh: Performed the experiments; Contributed reagents, materials, analysis tools or data; Wrote the paper.

Parvin Parvaei: Conceived and designed the experiments; Performed the experiments.

Freshteh Osmani: Analyzed and interpreted the data.

## Data availability statement

Data associated with this study has been deposited at birjand university medical science under the accession number 09105477044.

## Declaration of competing interest

The authors declare no conflict of interest.
